# Determinants of late antenatal care attendance among high parity women in Uganda: analysis of the 2016 Uganda demographic and health survey

**DOI:** 10.1186/s12884-023-06214-z

**Published:** 2024-01-05

**Authors:** Godfrey Tumwizere, Martin K. Mbonye, Patricia Ndugga

**Affiliations:** 1 School of Statistics and Planning, Makerere University, Kampala, Uganda; 2 Action 4 Health Uganda, Kampala, Uganda

**Keywords:** High parity, Late antenatal care, Uganda

## Abstract

**Background:**

Timely and adequate Antenatal Care (ANC) effectively prevents adverse pregnancy outcomes and is crucial for decreasing maternal and neonatal mortality. High-parity women (5 + children) are at higher risk of maternal mortality. Limited information on the late timing of ANC among this risky group continues to hamper Uganda’s efforts to reduce maternal mortality ratios and improve infant and child survival. This study aimed to determine factors associated with attendance of the first ANC after 12 weeks of gestation among high-parity women in Uganda.

**Methods:**

This study was based on nationally representative data from the 2016 Uganda Demographic and Health Survey. The study sample comprised 5,266 women (aged 15–49) with five or more children. A complementary log-log regression model was used to identify factors associated with late ANC attendance among high-parity women in Uganda.

**Results:**

Our findings showed that 73% of high parity women delayed seeking their first ANC visit. Late ANC attendance among high-parity women was associated with distance to the health facility, living with a partner, partner’s education, delivery in a health facility, and Desire for more children. Women who did not find the distance to the health facility when going for medical help to be a big problem had increased odds of attending ANC late compared to women who found distance a big problem (AOR = 1.113, CI: 1.004–1.234), women not living with partners (AOR = 1.196, 95% CI = 1.045–1.370) having had last delivery in a health facility (AOR = 0.812, 95% CI = 0.709–0.931), and women who desired to have another child (AOR = 0.887, 95% CI = 0.793–0.993) had increased odds compared to their counterparts.

**Conclusions:**

To increase mothers’ timely attendance and improve maternal survival among high-parity women in Uganda, programs could promote and strengthen health facility delivery and integrate family planning with other services such as ANC and postnatal care education to enable women to seek antenatal care within the recommended first trimester. This study calls for increased support for programs for education, sensitization, and advocacy for health facility-based deliveries. This could be done through strengthened support for VHT and community engagement activities.

## Background

Every day, approximately 830 women die from preventable causes related to pregnancy and childbirth [1], and almost all (99%) of these maternal deaths occur in developing countries. To achieve the Global Strategy for women, children, and adolescents’ health, antenatal care (ANC) should be put at the center of women’s health. ANC is care that a skilled healthcare professional provides to pregnant women and adolescent girls to ensure the best health conditions for both the mother and baby during pregnancy [1, 2]. ANC is concerned mainly with risk identification, prevention and management of pregnancy-related diseases, health education, and health promotion [3]. It offers an opportunity to promote healthy lifestyles for long-term health outcomes, establish delivery plans, and prepare the mother for parenting. To identify and address any issues and lower the risk of a stillbirth or neonatal mortality, the 2016 WHO recommendations on antenatal care for a good pregnancy experience suggested a woman see her health provider at least eight times throughout her pregnancy [2, 4]. ANC utilization is the frequency or the number of visits a pregnant woman makes to the antenatal clinic from the first visit until the end of pregnancy [5, 6]. This form of care for pregnant women is an essential pillar in the Safe Motherhood Programme, as the aim is to improve the outcome of pregnancy for both the mother and the fetus [7].

Studies have shown that ANC attendance improves maternal health by enabling women to prepare for delivery, understand and respond to pregnancy danger signs, thereby reducing maternal and infant morbidity and mortality [3, 6, 8–10]. The primary cause of pregnancy-related complications, ill health, and death is inadequate care of mothers during pregnancy and delivery, characterized by late attendance and underutilization of ANC services [5]. While any woman can develop complications during pregnancy and delivery, these complications can be prevented or treated before becoming life-threatening. All are managed through the timely utilization of ANC services [5]. Early ANC is crucial in detecting and treating some pregnancy complications and forms a reasonable basis for appropriate management during delivery. After childbirth [6, 11], Early ANC refers to initiating ANC as soon as possible after confirmation of pregnancy and or within the first 12 weeks of gestation. In contrast, late ANC is starting ANC after 12 weeks of gestation [4].

Globally, while 86% of pregnant women access ANC [4, 5], in developing countries, 11.2% of pregnant women overall have no ANC at all, and only 49.9% initiated ANC in the first trimester of pregnancy, while 43.3% and 6.8% postpone it until the second and third trimesters, respectively [12]. Generally, women in the wealthiest 20% of the population of their countries are more likely to receive ANC than poorer women, especially in the most deprived regions [13]. In areas with the highest maternal mortality rates, such as Sub-Saharan Africa (SSA) and South Asia, fewer women make the minimum recommended number of visits [14]. Although overall levels of antenatal care are relatively high across regions, there are disparities in terms of household wealth and urban or rural residence. In South Asia and sub-Saharan Africa, the urban-rural gap in coverage of the recommended minimum antenatal care visits exceeds 20% points in favor of urban areas [14].

In developing countries, including Uganda, the timing of first ANC visits decreases with the mother’s parity [5, 15]. High parity is a risk factor for adverse fetal outcomes. For instance, a study on the factors associated with maternal mortality in Ethiopia revealed that the risk of maternal morbidity, mortality, and other pregnancy-related complications is significantly higher among women with five or more children [6]. High parity is associated with preterm delivery, prenatal mortality, and neonatal morbidity.

Uganda has progressively increased ANC coverage in the previous decades. Almost all (97%) women of reproductive age receive Antenatal Care at least once [16]. The proportion of women receiving at least four Antenatal Care visits has increased in the previous decades (from 42% in 2001 to 60% in 2016). However, it is noteworthy that women who receive ANC in the first trimester have remained substantially low (29%) [16]. Most women’s late initiation of ANC compromises the gains from improving coverage. For this study, a pregnant woman who makes her first ANC Visit after 12 weeks of pregnancy is categorized under late ANC attendance.

## Conceptual framework

**Fig. 1:**
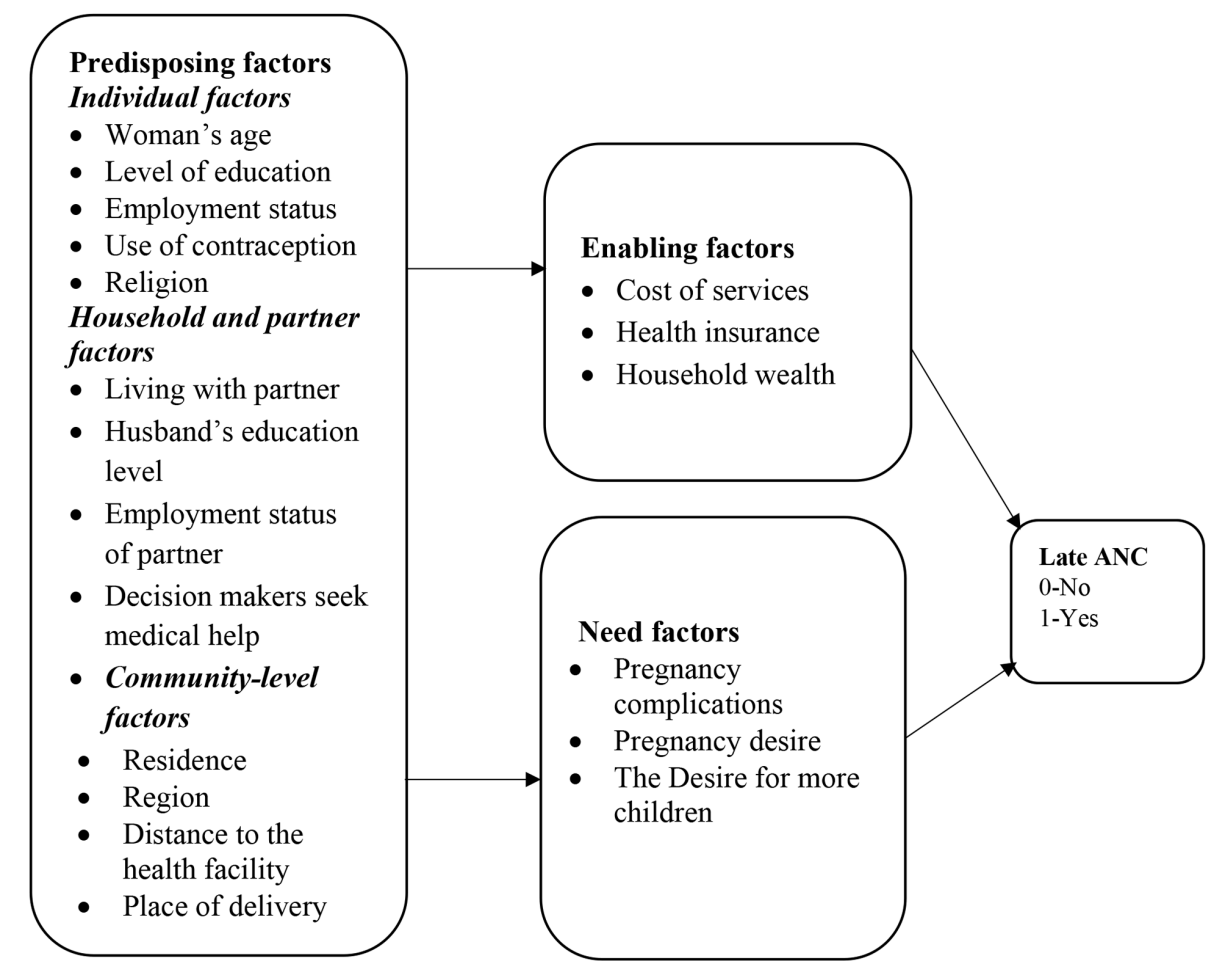
Conceptual framework of late ANC attendance. Adapted with modification from Tolera et al. (2020)

The conceptual framework for this study is shown in Fig. 1. Andersen’s Behavioral Model of health service utilization provided a relevant framework for understanding the determinants of Late ANC attendance among high-parity women in Uganda. This framework postulates that; the use of health care services, including Antenatal care, is a function of three sets of dynamics of Predisposing factors (the socio-cultural characteristics of individuals that exist before their need for health service), Enabling factors (the logistical aspects of obtaining care) and the Need factors (the most immediate cause of health service use) [17].

In this study, the predisposing factors were categorized into three levels; individual Levels, such as Woman’s age, Level of education, Employment status, Use of contraception, and Religion, while household factors included Household wealth, living with a partner, Husband’s education level, Employment status of partner and community level factors included Residence, Region, Distance to the health facility, Place of delivery. These factors can influence late ANC attendance in some ways. For example, older maternal age may lead some women to believe that early antenatal care attendance is not critical for optimal health outcomes due to confidence gained from previous pregnancies and births [18]. Contrary to this, younger women might have better knowledge of antenatal care services due to improved educational opportunities for women in recent years, leading to early ANC attendance. Women who use at least a family planning method have an increased knowledge base of antenatal care. During family planning counseling, women are given information on desired birth spacing and the importance of timely attendance to ANC; thus, women who have been using a method of contraception attend ANC early. A study on contraceptive-seeking behavior in women attending ANC in Nigeria revealed that women who were using the modern method of contraception had increased odds of attending Antenatal care [19]. Knowledge of ANC is associated with delayed initiation of ANC. A study on the late timing of ANC in Ethiopia revealed that those with low knowledge had nearly three times the likelihood of being late for ANC compared to women with a solid awareness of ANC [20]. Similarly, an analysis of factors associated with delayed initiation of ANC in Ethiopia revealed that knowledgeable women were less likely to delay their ANC initiation relative to non-knowledgeable women [20, 21].

Women’s Level of education can enhance individual autonomy and help women develop greater confidence and power to make decisions about their health, thereby influencing timely ANC attendance [22–27]. Furthermore, education can improve women’s knowledge and awareness of sexual and reproductive health issues, such as understanding that timely antenatal care contributes to positive maternal outcomes [24, 28] and hence make deliberate efforts to seek ANC early.

Enabling factors such as Cost of services, Health insurance, and household wealth influence the timing of ANC attendance. Household wealth enables women to seek ANC because women coming from wealthy households can afford the Cost of medical care. A study conducted in Ghana revealed that women with the wealthiest socioeconomic status had increased odds of early initiation of ANC visits [29]. Similarly, a study conducted in Western Ethiopia indicated that household income was significantly associated with late antenatal care entry by women [20, 30]. The role of wealth status and ANC service utilization has been reported in many studies [22, 31, 32]. These studies showed a significant positive relationship between antenatal care attendance and the wealth status of the women. Family wealth signifies the individual’s economic situation, thus influencing the use of Antenatal care services, as revealed in various studies [12, 33, 34].

Furthermore, need factors such as Pregnancy complications, Pregnancy desire, and Desire for more children also influence women’s timely attendance. These factors are known to pose risks to women and newborns and may affect women seeking early antenatal care. For example, women with a history of pregnancy complications are more likely to seek ANC early than women without complications. Studies on delayed initiation of ANC and associated factors revealed that women who experienced difficulties during pregnancy were less likely to delay their first ANC attendance than women who did not [3, 35]. Some women have reported that delayed initiation of ANC is due to the absence of pregnancy complications or limited knowledge about pregnancy complications. A study conducted in Tanzania contends that some women do not attend ANC until they experience pregnancy complications [36]. According to Rwandan research, women who did not know about pregnancy complications were less likely to use ANC services during the first trimester [35].

Also, women whose pregnancy was desired by the time they got pregnant and women who desired to have more children are more responsive to timely antenatal care attendance. In Ethiopia, a study found that, compared to women who had planned pregnancies, those who had unexpected pregnancies had 2.31 times greater odds of initiating ANC late [20]. Furthermore, a systematic review of delayed initiation of ANC in Ethiopia showed that women with intended pregnancies were less likely to delay their ANC initiation [37]. According to a study conducted on the timing and number of ANC visits among married and unmarried youths in Uganda, married children who did not want the pregnancy or who decided to have one later were 27% less likely to have their first ANC visit in the first trimester compared to those who wanted to become pregnant at that time [38]. Another study conducted in Uganda reported that mothers who wished to have pregnancy later and those who did not want it at all were less likely to initiate the first visit in the first trimester compared to counterparts who wanted it at the time they got pregnant [39]. Unplanned pregnancy was also identified as one factor influencing the late initiation of ANC in Kahama municipal of Tanzania [36] and South Africa, as women who had unplanned pregnancies were more likely to initiate ANC late [40].

Whereas every pregnant Woman is at risk of maternal mortality, high parity is a risk factor for adverse fetal outcomes; thus, high-parity women are at increased risk of maternal morbidity and mortality. Little is known about the determinants of late ANC attendance among this risky group in Uganda. Thus, this study aimed to investigate the determinants of late ANC attendance among high parity women in Uganda who had given birth within two years preceding the 2016 UDHS. The study attempted to answer the following research question: What are the determinants of late ANC attendance? We hypothesized that residing in rural areas, women living with their partners, women who have been using any method of contraception, women whose husbands are employed women who delivered their last previous child in a health facility, and women who perceive that distance from a facility does not hinder their access to health care are more likely to attend ANC late.

## Methods

### Study design and setting

This paper was based on secondary data from the 2016 Uganda Demographic and Health Survey. Authorization to use this data (accessed from The DHS Program website) was obtained upon providing a brief description of our study (The DHS Program 2018). The DHS used a two-stage cluster sampling design to generate a nationally representative sample of women aged 15–49 in sampled households. Enumeration areas were selected from a list of clusters in the first stage. In the second stage, households were selected. All women aged 15–49 from the households, including visitors who stayed in the household the night before the survey, were eligible for the interview. Details on the sampling procedure are described in the UDHS final report [16]. This analysis used the women’s individual recode data file.

### Study population

**Fig. 2:**
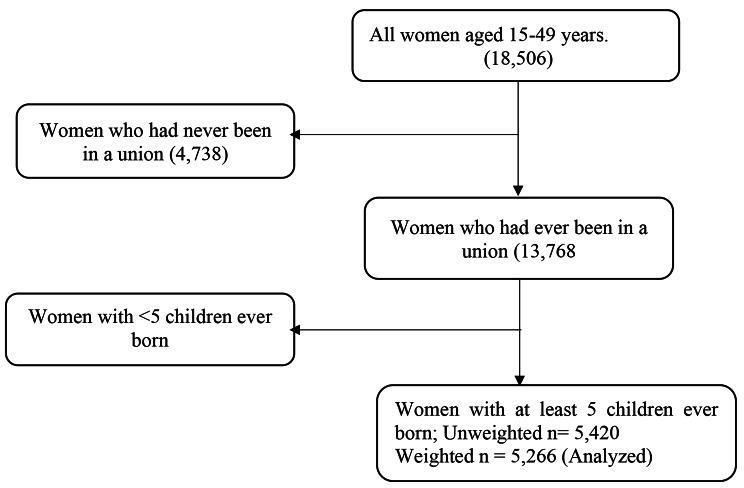
Sample selection criteria

Figure 2 shows the sample selection criteria used for this study. The study sample comprised women with at least five children born before the 2016 UDHS survey. Our analysis of late ANC attendance was limited to the women who had ever been in a union. The final sample comprised 5266 high-parity women.

### Outcome variable

In the DHS Women’s Questionnaire, all women who gave birth in the five years preceding the survey were asked about the number of children they had ever given birth to. Women were also asked about the timing of their first antenatal care reported in weeks.

Therefore, the outcome variable for this study was “Late antenatal care attendance,” defined as having attended the first antenatal care after 12 weeks of pregnancy. The outcome variable is late antenatal attendance with a binary outcome. All high-parity women who reported their first ANC at four or more months were coded 1 = “late ANC”; otherwise, they were coded 0 = “Early ANC.”

### Independent variables

The independent variables included in this study were the Woman’s age, region, residence, Woman’s Level of education, religion, wealth quintile, survival status of last birth, method of contraception, pregnancy wanted at conception, Place of delivery, mode of delivery, permission to seek medical help, Cost of medical care, distance to the health facility, health insurance, living (residing) with husband/partner, husband’s Desire for children, husband/partner’s; age, education level, occupation, decision-maker for Woman’s health care and Woman’s employment status. Woman’s age was grouped into three categories: 20–29, 30–39, and 40–49. The age group 15–19 had only a few cases and were regrouped with the age group 20–29. Women’s level of education was recorded in three categories. All the other categories remained as they are in the DHS, apart from the categories of secondary education and Higher education, which combined into a category called “Secondary +). This was also the case for the variable on the partner’s Level of education. The DHS data had many categories, but these were combined into five major categories. Women who reported their religious affiliation as having no religion were combined with the other category. Anglicans, Catholics, Muslims, and other Christian religious groups such as Orthodox, SDA, and Born-again were combined into “Other Christians. “The 14 regions in the DHS were regrouped into four major regions (Central, Eastern, Northern, and Western). The central region comprises Kampala, South Buganda, and North Buganda. The eastern region includes Busoga, Bukedi, Bugisu, Teso, and Karamoja. Lango, Acholi, and West Nile were combined into the Northern region, while Bunyoro, Tooro, Ankole, and Kigezi formed the Western region. The variable of contraceptive use was recorded from the current contraceptive use by method variable in the DHS to generate two categories. All the women who had never used any method were coded as “Not using,” while those who had used any method were coded as “Using. “The variable on the timing (in months) of the first ANC visit was recoded to generate categories of “Early ANC” and “Late ANC. “All first ANC visits after three months were combined with “Late ANC visits. “The variable Place of last delivery was recoded into three categories “home,” which combined all home-based deliveries reported by the Woman, “health facility,” which comprised both public and private health facility-based deliveries; and the other category for all the others. The husband’s/partner’s occupation was re-categorized into; “Not-working,” “Working for Others,” and “Self-employed.”

### Statistical analysis

Data analysis was done using STATA software (version 13.0). Data was weighted to ensure the representativeness of the study population. The analyses were done at three levels.

First, the frequency and percent distribution of the characteristics of high-parity women were run and presented for both dependent and independent variables. Second, cross tabulations with chi-square tests were run for late antenatal attendance and each explanatory variable. Pearson’s chi-square test was performed to measure the significance of associations with late ANC attendance among high-parity women at 5% level of significance. All variables with a ρ-value less than or equal to 0.05 were considered to have a significant association with late ANC attendance. These were automatically considered for further analysis at the multivariate Level. In addition to the variables significantly associated with late ANC attendance at the bivariate Level, those that were not significant but whose *p*-value was less or equal to 0.1 and were supported by literature to have a significant association with late ANC attendance were included in the multivariate model.

At the multivariate Level, since late ANC attendance was measured on a binary scale (0 = early ANC and 1 = late ANC), the logistic, probit, and complementary log-log regression models were all potential models for the study; thus, tests were done to identify the most appropriate model for the data. To determine the model that best suited the data, the Akaike Information Criterion (AIC) and link test tested each of the three models for the goodness of fit. First, the Akaike Information Criterion (AIC) assessed each model for appropriateness. When using the AIC, the model with the lowest information criterion value is considered the most appropriate for analysis. All the models had an almost equal value of AIC. Thus, the complementary log-log model was selected for the study. Secondly, a link test was performed for each of the three models to determine if the squared term (independent variable) does not have more explanatory power (no link error) in the model. A model is appropriate if the squared term hatsq (ρ > 0.05) is insignificant. The complementary log-log model was selected because it fit this criterion. Thus, this model was fitted to assess the impact of each statistically significant independent variable (ρ-value < 0.05) on the outcome variable (late ANC attendance).

## Results

Results presented in Table 1 show that out of the weighted sample of 5266 women, almost half (49%) of the high-parity women were aged 30–39 years and older, while 42% were 40 years and older. Also, most (66%) of the high parity women had attained primary education, 84% were rural residents, 85% were currently working, and 62% were not using a contraceptive method. The findings in the table also reveal that 46% of the high-parity women said they intended to get pregnant then. Regarding religion, the results in Table 1 show that 4 in 10 high parity women were affiliated with the Catholic faith while 32% were Anglicans. Furthermore, 39% of the women were from the Eastern region of Uganda, 25% were from the Western region, and 24% were from the Central region.

**Table 1:** Distribution of High Parity women by selected characteristics, UDHS 2016

Characteristic	Frequency (n = 5266)	Percent (%)
**Maternal age (years)**		
20–29	501	9.5
30–39	2569	48.8
40–49	2196	41.7
**The highest education level attained**		
No education	1,157	22.0
Primary	3,473	66.0
Secondary+	636	12.1
**Woman’s Place of residence**		
Urban	862	16.4
Rural	4404	83.6
**Women’s current working status**		
Not working	800	15.2
Working	4467	84.8
**Contraceptive use**		
Not using	3,236	61.5
Using	2,030	38.6
**Pregnancy intention (n = 3420)**		
Then	1590	46.0
Later	925	26.8
No more	939	27.2
**Religion**		
Anglican	1,698	32.2
Catholic	2,103	39.9
Muslim	630	12.0
Pentecostals	782	14.8
Other	53	1.0
**Region**		
Central	1,240	23.6
Eastern	2,056	39.0
Northern	673	12.8
Western	1,297	24.6
**Getting money needed for treatment**		
Big problem	2,901	55.1
Not a big problem	2,365	44.9
**Distance to the health facility for treatment**		
Big problem	2,419	45.9
Not a big problem	2,847	54.1
**Place of delivery (n = 3420)**		
Home	1,109	32.4
Health facility	2,242	65.6
Other	69	2.0
**Household wealth**		
Lower	2,366	44.9
Middle	1,196	22.7
High	1,704	32.4
**Whether the respondent was living with a partner (n = 4332)**		
Living with partner	3647	84.2
Staying elsewhere	685	15.8
**Covered by health insurance**		
No	5,209	98.9
Yes	57	1.1
**Husband/partner’s education level (n = 4332)**		
No education	409	9.4
Primary	2,651	61.2
Secondary+	1,165	26.9
Don’t know	107	2.5
**Husband/partner’s occupation (n = 4,318)**		
Not working	211	4.9
Working for others	2154	49.9
Self-employed	1954	45.3
**The Desire for more children**		
No more	3498	66.4
Have another	1087	20.6
Undecided	187	3.5
Sterilized/declared infecund	495	9.4
**Decision on respondent’s health care (n = 4,331)**		
Respondent	1485	34.3
Joint	1806	41.7
Other	1041	24.0

Note: *The variations in totals are because some of the questions were asked to women who had given birth recently, while the others were for all women*

The results also indicate that more than half (55%) of the high-parity women reported that getting the money needed for their treatment was a big problem. In comparison, 54% said the distance to the health facility for treatment was a big problem. In terms of Place of delivery, more than 6 in 10 high-parity women said that their most recent child delivery had taken Place in a health facility. The table also indicates that 45% of the women were from the poor wealth category of households, 23% were from the middle quintile, and 22% were from the wealthy class. Table 1 reports that most (84%) of the high-parity women said they were living with their partners.

Additionally, almost all (99%) women were not covered by health insurance. The results also show that slightly more than 6 in 10 (61%) of the women said their husbands/partners had attained primary education, 50% said their partners were working for others, and 45% reported that their partners were self-employed. Also, 67% of the women said they did not desire more children at the time of the survey, and 42% reported that they jointly decided on healthcare matters with their partners. Table 1 shows the details.

**Fig. 3:**
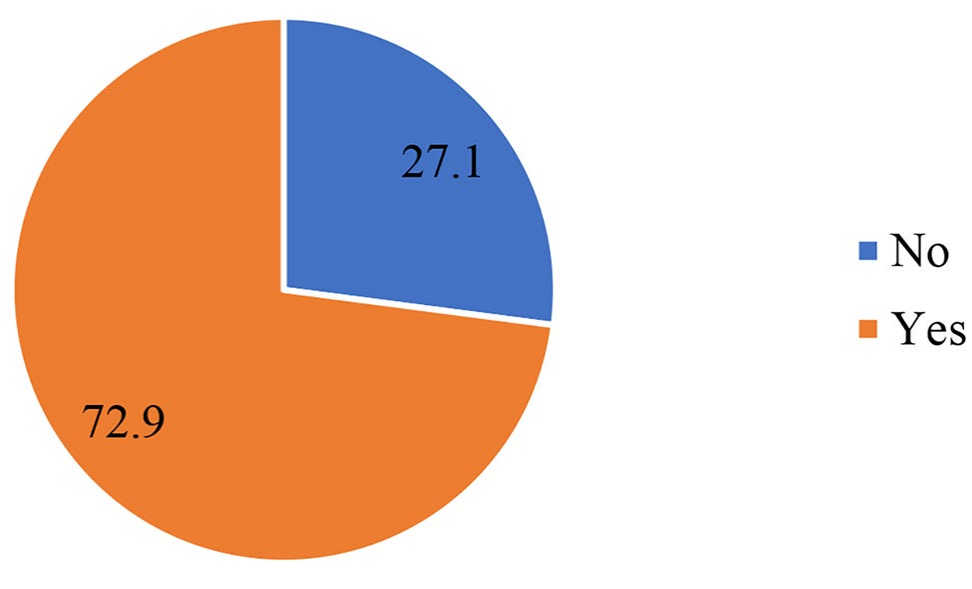
Percentage distribution of ANC attendance among high-parity women

The results in Fig. 3 indicate that the majority (73%) of the high parity women reported that the timing of their first ANC attendance was late (after the first trimester).

### Bivariate analysis of factors associated with late ANC attendance

Results from Table 2 show no significant association between the Woman’s age and late ANC attendance (**ρ** > 0.05). The results indicate that across all the age groups, at least 7 in 10 high-parity women reported attending ANC late. Similarly, more than 7 in 10 of the women in each category of education level attained had attended ANC late. Education level reached, and late ANC attendance had no significant relationship (**ρ** > 0.05). Similarly, the table shows that while 71% and 73% of the high-parity women who resided in urban and rural areas, respectively, reported late ANC attendance, the association between Place of residence and late ANC attendance was not significant at the 5% level of significance. The results in the table also indicate that current working status (**ρ** = 0.895), pregnancy intention (**ρ** = 0.433), religious affiliation (**ρ** = 0.340), getting the money needed for treatment (**ρ** = 0.169), household wealth (ρ = 0.590), health insurance coverage (**ρ** = 0.365) and decision-making regarding respondents’ health care were not significantly associated with late ANC attendance. Table 2 shows more details.

**Table 2:** Percentage distribution of high parity women by late ANC attendance

Characteristic	Late ANC attendance	Frequency (n = 5266)	*P*-value
**Age (years)**			
20–29	350 (69.8)	501	0.479
30–39	1860 (72.4)	2569	
40–49	1628 (74.8)	2196	
**Education level attained**			
No education	830 (71.7)	1,157	0.741
Primary	2535 (73.0)	3,473	
Secondary+	471 (74.0)	636	
**Place of residence**			
Urban	611 (70.9)	862	0.425
Rural	3224 (73.2)	4404	
**Current working status**			
Not working	580 (72.5)	800	0.846
Working	3256 (72.9)	4467	
**Current use of contraception**			
Not using	2338 (74.2)	3,236	0.083*
Using	1439 (70.9)	2,030	
**Wanted pregnancy when became pregnant**			
Then	1156 (72.7)	1590	0.543
Later	663 (71.7)	925	
No more	697 (74.3)	939	
**Religious affiliation**			
Anglican	1234 (72.7)	1,698	0.327
Catholic	1524 (72.5)	2,103	
Muslim	441 (70.0)	630	
Other Christians	597 (76.3)	782	
Other	42 (78.7)	53	
**Region**			
Central	941 (75.9)	1,240	0.104
Eastern	1468 (71.4)	2,056	
Northern	465 (69.1)	673	
Western	971 (74.9)	1,297	
**Distance to the health facility**			
Big problem	1722 (71.2)	2,419	0.069*
Not a big problem	2115 (74.3)	2,847	
**Getting money needed for treatment**			
Big problem	2080 (71.7)	2,901	0.136
Not a big problem	1757 (74.3)	2,365	
**Place of delivery (n = 3420)**			
Home	866 (78.1)	1,109	0.004**
Health facility	1576 (70.3)	2,242	
Other	52 (75.1)	69	
**Household wealth index**			
Lower	1706 (72.1)	2,366	0.652
Middle	879 (73.5)	1,196	
High	1256 (73.7)	1,704	
**Living with a partner (n = 4332)**			
Yes	2647 (72.4)	3647	0.026**
No	532 (77.7)	685	
**Covered by health insurance**			
No	3803 (73.0)	5,209	0.369
Yes	36 (63.4)	57	
**Husband/partner’s education (n = 4332)**			
No education	271 (66.2)	409	0.007**
Primary	1991 (75.1)	2,651	
Secondary+	823 (70.6)	1,165	
Don’t know	88 (82.0)	107	
**Husband/partner’s occupation**			
Not working	138 (65.2)	211	0.078*
Working for others	1568 (72.8)	2154	
Self-employed	1458 (74.6)	1954	
**The Desire for more children**			
No more	2610 (74.6)	3498	0.083*
Have another	761 (70.0)	1087	
Undecided	135 (72.4)	187	
Sterilized	330 (66.7)	495	
**Decision maker on respondent’s health care**			
Respondent	1087 (73.2)	1485	0.874
Joint	1331 (73.7)	1806	
Other	755 (72.5)	1041	

The values with * in the p-value column indicate p < 0.1, while ** show p < 0.05

The results in Table 2 indicate that at the 5% level of significance, only Place of delivery (ρ = 0.003), living with a partner (*p* = 0.026), and husband/partner’s education (ρ = 0.007) were significantly associated with late ANC attendance. The results, however, show that current use of contraception (ρ = 0.084), region (*p* = 0.083), distance to health facility (ρ = 0.087), husband/partner’s occupation (*p* = 0.078), and the Woman’s Desire for more children (ρ = 0.070) were only significant at the 10% level of significance. Thus, these were considered for multivariate analysis.

### Multivariate analysis

Table 3 shows the results of a multivariate complementary log-log of late antenatal care attendance. Although the bivariate analysis indicated that women’s age was not significantly associated with early postnatal care utilization, this variable was considered for inclusion in the Complementary log-log model based on previous literature.

**Table 3:** Regression of factors associated with late Antenatal care attendance

Characteristic	Frequency (n = 5266)	Late ANC attendance	Adjusted OR	95% CI	*P*-value
**Current use of contraception**					
Not using	3,236	2338 (74.2)	1.000		
Using	2,030	1439 (70.9)	0.911	0.113–0.812	0.113
**Distance to the health facility**					
Big problem	2,419	1722 (71.2)	1.000		
Not a big problem	2,847	2115 (74.3)	1.125	1.016–1.245	***0.023***
**Place of delivery**					
Home	1,109	866 (78.1)	1.000		
Health facility	2,242	1576 (70.3)	0.800	0.700-0.914	***0.001***
Other	69	52 (75.1)	0.911	0.626–1.328	
**Living with partner**					
Yes	3647	3803 (73.0)	1.000		
No	685	36 (63.4)	1.186	1.034–1.361	***0.015***
**Husband/partner’s education**					
No education	409	138 (65.2)	1.000		
Primary	2,651	1568 (72.8)	1.305	1.072–1.589	***0.008***
Secondary+	1,165	1458 (74.6)	1.184	0.957–1.466	0.120
Don’t know	107	138 (65.2)	1.648	1.117–2.433	***0.012***
**Husband/partner’s occupation**					
Not working	211	138 (65.2)	1.000		
Working for others	2154	1568 (72.8)	1.205	0.954–1.521	0.117
Self-employed	1954	1458 (74.6)	1.287	1.025–1.617	***0.030***
**The Desire for more children**					
No more	3498	2610 (74.6)	1.000		
Have another	1087	761 (70.0)	0.886	0.792–0.992	***0.036***
Undecided	187	135 (72.4)	0.914	0.689–1.212	0.532
Sterilized	495	330 (66.7)	0.810	0.622–1.053	0.116

***Bold italics indicate significant values at α = 0.05 (p < 0.05)***

The results in Table 3 show that late ANC attendance by high parity women was significantly associated with distance to the health facility to access health services, Place of delivery, not living with a partner, husband/partner’s Level of education, having a self-employed partner and the Desire to have another child. On the other hand, the current use of contraception was not significantly associated with late ANC attendance.

Specifically, Table 3 indicates that high-parity women who reported that the distance to the health facility when going for their medical help was not a big problem had increased odds (AOR = 1.113, CI: 1.004–1.234) of late ANC attendance compared to their counterparts who said that the distance to the health facility was a big problem. Relatedly, high parity women who were not living with a partner were associated with an increased likelihood (AOR = 1.186, 95% CI = 1 1.034–1.361) of late ANC attendance compared to those living with a partner. High parity women who said that their partners/husbands had attained primary education had high odds (AOR = 1.305, 95% CI = 1.072–1.589) of attending ANC late compared to those whose partners had no education. Similarly, high parity women who reported not knowing their partner’s/husband’s education level were 1.6 times (AOR = 1.648, 95% CI = 1.117–2.433) as likely to attend ANC late relative to their counterparts whose partners had no education. The findings also showed that women whose partners were self-employed were more likely to initiate ANC late than their counterparts whose partners were not working.

On the other hand, the findings also indicate that high-parity women who reported their last delivery had taken Place in a health facility were less likely (AOR = 0.800, 95% CI = 0.700-0.914) to attend ANC late compared to those who reported that their last Place of delivery was home. The findings also reveal that women who desired to have another child had reduced odds (AOR = 0.886, 95% CI = 0.792–0.992) of late ANC attendance compared to their counterparts who wanted no more children.

## Discussion

The study aimed to determine the factors associated with late ANC attendance among high-parity mothers in Uganda. The study found that most (73%) women sought antenatal care late. The World Health Organization recommends that pregnant women start the first ANC in the first trimester of pregnancy, which helps to ensure the best care and health outcomes for women and their fetuses. However, this study’s findings imply that most women miss vital interventions recommended for receiving a package throughout their pregnancy.

In this study, women who do not stay with their husbands/partners had increased odds of attending ANC later than those staying with their husbands. This finding may imply that the women who did not live with their partners had limited/inadequate support. Men’s role in reproductive health is critical in timely attendance to ANC. Partner support is essential for ensuring timely access to services, including ANC. This study partly agrees with those of a study conducted in South Africa, which reported a significant association between late presentation for ANC in rural communities and being married (Ebonwu et al., 2018) but disagrees with the findings of Tesfaye et al. [21], who demonstrated that there was no significant association between marital status and delayed initiation of ANC. Also, women living with their husbands have the advantage of being reminded to seek ANC and get support in terms of transport or the cost of care. This finding is similar to other studies by [11, 41, 42].

Further studies in Rwanda, Lesotho, and Tanzania also revealed that single women initiate ANC attendance late compared to women living with their spouses [43, 44]. The findings also showed that women whose partners were self-employed were more likely to start ANC late than their counterparts whose partners were not working. This may be because self-employed men may find excuses for not accompanying their partners to ANC. This, however, needs further investigation.

Women using at least a method of contraception are less likely to attend ANC late than those without contraception. This finding is in agreement with the study carried out in Nigeria, where women who were using modern methods of contraception had increased odds of attending ANC early [45]. Women using a modern method of contraception are exposed to health education, which includes timely attendance of ANC. Similar findings were revealed in India, where women who had used modern contraceptives had increased odds of attending ANC early [46].

The findings indicated that high-parity women who reported that the distance to the health facility when going for medical help was not a big problem had increased odds of late ANC attendance compared to their counterparts who said that the distance to the health facility was a big problem. This finding is partly in line with other studies [11] which reported long distance to health facilities as one of the critical factors for late initiation of ANC. The finding agrees with; those of a qualitative study among pregnant women attending ANC in Tanzania, which revealed that distance from people’s settlement to the health facility was among the major hindrances to early initiation of ANC [36]. The findings are also in line with those of a study in Ethiopia, which revealed that women who paid for transportation to get to ANC services had a 72% higher chance of scheduling ANC services late than mothers who did not [20, 47].

The study found that high-parity women whose partners/husbands had attained primary education had increased odds of attending ANC late compared to those whose partners had never attained education. This finding may be attributable to limited knowledge about ANC and the correct timing among partners who do not have adequate education. The result implies that there is a need for increased sensitization of high-parity women and their partners regarding ANC matters. Women whose husbands attained primary education had increased odds of attending ANC late compared to those whose husbands had no education. Educated husbands appreciate the importance of antenatal care and thus encourage their women to seek antenatal care early [48]. This finding is partly in line with studies conducted in Ethiopia [21] and Tanzania [36], which reported that women whose husbands had formal education were less likely to delay their first prenatal appointment than women whose husbands had no formal education. However, this finding contradicts the findings in Nepal, where women whose partners were uneducated were found to attend antenatal care early [49, 50].

High-parity women who reported that their last delivery had taken Place in a health facility were less likely to attend ANC late compared to those who reported that their last Place of delivery was at home. This finding may partly be because mothers who deliver at a health facility receive health education that may motivate and encourage them to attend ANC in a timely. This is partly in agreement with other studies [11, 48, 51, 52], which reported that women who gave birth at a health facility were more likely to attend ANC early compared to those who gave birth at home.

The findings also revealed that women who desired to have another child had reduced odds of late ANC attendance compared to their counterparts who desired no more children. This is partly because women with such desires are motivated to ensure safety during pregnancy, delivery, and post-delivery. The motivation to have a healthy child may explain why the women who desire another child are less likely to attend ANC. This is partly in line with the findings of previous studies in Myanmar [36] (Aung et al., 2016), Ethiopia [21], Tanzania [36], and in South Africa (Ebonwu et al., 2018), Uganda [38] which all observed reduced odds of late ANC initiation among women whose desires were to have a child soon.

This study analyzed data from the Uganda Demographic Health Survey, a nationally representative survey of women. This provides a national perspective on the findings of the study. By focusing on high-parity women who are also reportedly associated with risks of pregnancy complications and maternal mortality, this study attempted to identify factors that are related to late timing of ANC attendance among this risky group of women. The findings can thus help in improved targeting of health interventions that aim to improve maternal and child health outcomes. It is noteworthy, however, that this study was cross-sectional and presents associations between the selected variables and late ANC initiation among high-parity women. Therefore, this study’s findings do not imply causes of delay in attending ANC. The methodology adopted is limited to assessing associations between the selected variables. In addition, being a secondary analysis, the study acknowledges that some important variables, such as accessibility of healthcare services and affordability, could not have been explained in detail by the survey. The study does not provide in-depth explanations of late ANC attendance and thus calls for a qualitative investigation. Nevertheless, the findings highlight areas needing more research through primary data studies.

### Limitations

This study used secondary data from demographic and health surveys, which do not capture data on perceptions about antenatal care attendance among high-parity women in Uganda that could influence the timing of the first ANC visit. In the DHS survey, some questions were explicitly directed toward women who had recently given birth, while others were intended for all women. This distinction resulted in varying sample sizes for different questions, introducing a level of data disproportion. Also, being cross-sectional, this study could only measure associations rather than make causal inferences.

## Conclusions

Most high-parity women were found to attend their first ANC after the first trimester of their pregnancy. Late ANC attendance was associated with being from the Northern region, finding distance to a health facility to be a big problem, not living with a partner, having a partner who had attained a primary Level of education, delivering from a health facility, and Desire for another child. The study found no evidence in the data to reject the hypotheses that high-parity women residing in rural areas are more likely to attend ANC late compared to ones in urban areas and That parity women who have been using a method of contraception are more likely to attend ANC late compared to ones who do not use a method of contraception. Findings supported the hypotheses that high-parity women who have been using any method of contraception are more likely to attend ANC late compared to ones who do not use a method of contraception. High-parity women whose husbands are employed are more likely to attend ANC late than those whose husbands are unemployed.

On the other hand, the study rejects the hypothesis that high-parity women who do not live with their partners are less likely to attend ANC late compared to women who live with their partners and concludes that high-parity women who do not live with their partners are more likely to attend ANC late compared to their counterparts who live with their partners. Similarly, the hypothesis that high-parity women who delivered their last child from a healthy facility are more likely to attend ANC late compared to ones who delivered from home was rejected. The study concludes that high-parity women who deliver their children from a health facility are less likely to initiate ANC attendance late. The study recommends that Uganda’s policymakers and program implementers work towards formulating policies and programs to address factors significantly reducing late ANC attendance. For example, integrating family planning with other services, such as ANC and postnatal care education, could enable women to seek antenatal care within the recommended first trimester. This would not only reduce late ANC attendance but would go a long way in improving maternal and child health outcomes.

The study findings highlight the need to increase health facility-based deliveries. This study calls for increased support for programs for education, sensitization, and advocacy for health facility-based deliveries. This could be done through strengthened support for VHT and community engagement activities.

There is also a need to target men with health education programs so that they can support their partners in health-seeking behavior. Men should be sensitized and educated about health issues and programs to address health problems such as poor maternal outcomes, which could easily be prevented through their active participation. Male involvement efforts should be strengthened. There is a need to carry out a qualitative survey to assess how and why social, cultural, economic, and community factors influence the late initiation of ANC among high-parity women. This would provide a more in-depth investigation of the factors from the women’s and community perspectives.

This study looked at determinants of late ANC attendance using data from recent demographic and health surveys. Therefore, there is also a need to carry out a trend analysis of factors associated with late ANC attendance from 2000 to 2016 to identify factors that have persistently kept late ANC attendance high.

## Data Availability

The dataset used for this study is publicly available through the link http://www.dhsprogram.com/data/dataset_admin/login_main.cfm.

## Abbreviations

ANC: Antenatal care
UDHS: Uganda Demographic and Health Survey
WHO: World Health Organization

## References

[1] World Health Organization. WHO recommendations on antenatal care for a positive pregnancy experience. World Health Organization; 2016. 28079998

[2] Weeks A, Temmerman M (2016). New WHO antenatal care model–quality worth paying for?. The Lancet.

[3] Tolefac PN, Halle-Ekane GE, Agbor VN, Sama CB, Ngwasiri C, Tebeu PM (2017). Why do pregnant women present late for their first antenatal care consultation in Cameroon?. Matern Health Neonatol Perinatol.

[4] Adow I, Mwanzo I, Agina O, Wanzala P, Kariuki J (2020). Uptake of antenatal care services among women of reproductive age in Mandera County, Kenya. Afr J Health Sci.

[5] Moller AB, Petzold M, Chou D, Say L. Early antenatal care visit: a systematic analysis of regional and global levels and trends of coverage from 1990 to 2013. Lancet Glob Health [Internet]. 2017;5(10):e977–83. 10.1016/S2214-109X(17)30325-X. 10.1016/S2214-109X(17)30325-X PMC5603717 28911763

[6] Wilunda C, Quaglio G, Putoto G, Takahashi R, Calia F, Abebe D et al. Determinants of utilization of antenatal care and skilled birth attendant at delivery in South West Shoa Zone, Ethiopia: A cross-sectional study. Reprod Health [Internet]. 2015;12(1):1–12. 10.1186/s12978-015-0067-y. 10.1186/s12978-015-0067-y PMC4592558 26432298

[7] Grum T, Brhane E (2018). Magnitude and factors associated with late antenatal care booking on a first visit among pregnant women in public health centers in the central zone of Tigray Region, Ethiopia: a cross-sectional study. PLoS ONE.

[8] Adedokun ST, Yaya S (2020). Correlates of antenatal care utilization among women of reproductive age in sub-saharan Africa: evidence from multinomial analysis of demographic and health surveys (2010–2018) from 31 countries. Archives of Public Health.

[9] Fekadu GA, Kassa GM, Berhe AK, Muche AA, Katiso NA (2018). The effect of antenatal care on institutional delivery service and postnatal care use in Ethiopia: a systematic review and meta-analysis. BMC Health Serv Res.

[10] Owolabi OO, Wong KLM, Dennis ML, Radovich E, Cavallaro FL, Lynch CA et al. Comparing the use and content of antenatal care in adolescent and older first-time mothers in 13 countries of West Africa: a cross-sectional analysis of Demographic and Health Surveys. Lancet Child Adolesc Health [Internet]. 2017;1(3):203–12. 10.1016/S2352-4642(17)30025-1. 10.1016/S2352-4642(17)30025-1 30169169

[11] Kawungezi PC, AkiiBua D, Aleni C, Chitayi M, Niwaha A, Kazibwe A (2015). Attendance and utilization of antenatal care (ANC) services: a multi-center study in upcountry areas of Uganda. Open J Prev Med.

[12] Jiwani SS, Amouzou A, Carvajal-Aguirre L, Chou D, Keita Y, Moran AC (2020). Timing and number of antenatal care contacts in low-and middle-income countries: analysis in the countdown to 2030 priority countries. J Global Health.

[13] Arsenault C, Jordan K, Lee D, Dinsa G, Manzi F, Marchant T (2018). Equity in antenatal care quality: an analysis of 91 national household surveys. Lancet Glob Health.

[14] Goli S, Nawal D, Rammohan A, Sekher T, v, Singh D (2018). Decomposing the socioeconomic inequality in utilization of maternal health care services in selected countries of South Asia and Sub-saharan Africa. J Biosoc Sci.

[15] Mwinyikione S (2016). A quantitative study on the determinants of utilization of skilled birth attendance, Bamba division, Kilifi. Int J Community Med Public Health.

[16] ICF U. Uganda demographic and health survey 2016. Kampala, Uganda: UBOS and ICF. 2018.

[17] Hirshfield S, Downing MJ, Horvath KJ, Swartz JA, Chiasson MA (2018). Adapting Andersen’s behavioral model of Health Service Use to examine risk factors for Hypertension among U.S. MSM. Am J Mens Health.

[18] Ayanore MA, Pavlova M, Groot W (2016). Focused maternity care in Ghana: results of a cluster analysis. BMC Health Serv Res.

[19] Ehwarieme TA, Iriafen LZ. Knowledge, attitude, and practice of Family Planning among Women Attending Maternal and Child Health Clinic NIFOR Benin, Edo State, Nigeria.

[20] Wolde HF, Tsegaye AT, Sisay MM (2019). Late initiation of antenatal care and associated factors among pregnant women in Addis Zemen primary hospital, South Gondar, Ethiopia. Reprod Health.

[21] Tesfaye G, Loxton D, Chojenta C, Semahegn A, Smith R (2017). Delayed initiation of antenatal care and associated factors in Ethiopia: a systematic review and meta-analysis. Reproductive Health.

[22] Akibu M, Tsegaye W, Megersa T, Nurgi S. Prevalence and determinants of complete postnatal care service utilization in northern Shoa, Ethiopia. J Pregnancy. 2018;2018. 10.1155/2018/8625437 PMC6112074 30186633

[23] Somefun OD, Ibisomi L (2016). Determinants of postnatal care non-utilization among women in Nigeria. BMC Res Notes.

[24] Dahiru T, Oche OM. Determinants of antenatal care, institutional delivery and postnatal care services utilization in Nigeria. Pan Afr Med J. 2015;22(1). 10.11604/pamj.2015.21.321.6527 PMC4633744 26587168

[25] Aliyu AA, Dahiru T. Predictors of delayed Antenatal Care (ANC) visits in Nigeria: secondary analysis of 2013 Nigeria Demographic and Health Survey (NDHS). Pan Afr Med J. 2017;26. 10.11604/pamj.2017.26.124.9861 PMC5429423 28533847

[26] Dahiru T, Oche OM. Determinants of antenatal care, institutional delivery and postnatal care services utilization in Nigeria. Pan Afr Med J. 2015;21(1). 10.11604/pamj.2015.21.321.6527 PMC4633744 26587168

[27] Regassa N. Antenatal and postnatal care service utilization in southern Ethiopia: a population-based study. Afr Health Sci. 2011;11(3). PMC3260999 22275929

[28] Acharya D, Khanal V, Singh JK, Adhikari M, Gautam S (2015). Impact of mass media on the utilization of antenatal care services among women of rural community in Nepal. BMC Res Notes.

[29] Manyeh AK, Amu A, Williams J, Gyapong M (2020). Factors associated with the timing of antenatal clinic attendance among first-time mothers in rural southern Ghana. BMC Pregnancy Childbirth.

[30] Tola W, Negash E, Sileshi T, Wakgari N (2021). Late initiation of antenatal care and associated factors among pregnant women attending antenatal clinic of Ilu Ababor Zone, Southwest Ethiopia: a cross-sectional study. PLoS ONE.

[31] Titaley CR, Dibley MJ, Roberts CL. Factors associated with underutilizing antenatal care services in Indonesia: results of Indonesia demographic and Health Survey 2002/2003 and 2007. BMC Public Health. 2010;10. 10.1186/1471-2458-10-485 PMC2933719 20712866

[32] Y T, T G, I G, K E, H L, MS S. Determinants of antenatal and delivery care utilization in Tigray region, Ethiopia: A cross-sectional study. Int J Equity Health [Internet]. 2013;12(1):1–10. Available from: https://equityhealthj.biomedcentral.com/articles/10.1186/1475-9276-12-30, 10.1186/1475-9276-12-30. 10.1186/1475-9276-12-30 PMC3658893 23672203

[33] Fagbamigbe AF, Idemudia ES (2017). Wealth and antenatal care utilization in Nigeria: policy implications. Health Care Women Int.

[34] Sunil TS, Spears WD, Hook L, Castillo J, Torres C (2010). Initiation of and barriers to prenatal care use among low-income women in San Antonio, Texas. Matern Child Health J.

[35] Kpienbaareh D, Kofinti RE, Konkor I, Amoak D, Kansanga MM, Luginaah I (2022). Knowledge of pregnancy Complications and utilization of antenatal care services in Rwanda. Int J Health Plann Manage.

[36] Bakari H, Mahiti GR (2022). Factors for late initiation of Antenatal Care in Kahama Municipal, Tanzania. Eur J Clin Med.

[37] Tesfaye G, Loxton D, Chojenta C, Semahegn A, Smith R (2017). Delayed initiation of antenatal care and associated factors in Ethiopia: a systematic review and meta-analysis. Reprod Health.

[38] Agaba P, Magadi M, Onukwugha F, Misinde C (2021). Factors associated with the timing and number of Antenatal care visits among unmarried compared to married youth in Uganda between 2006 and 2016. Soc Sci.

[39] Kisuule I, Kaye DK, Najjuka F, Ssematimba SK, Arinda A, Nakitende G (2013). Timing and reasons for coming late for pregnant women’s first antenatal care visit at Mulago Hospital, Kampala, Uganda. BMC Pregnancy Childbirth.

[40] Ebonwu J, Mumbauer A, Uys M, Wainberg ML, Medina-Marino A (2018). Determinants of late antenatal care presentation in rural and peri-urban communities in South Africa: a cross-sectional study. PLoS ONE.

[41] Komuhangi G (2020). Socio-demographics and late antenatal care seeking behavior: a cross-sectional study among pregnant women at Kyenjojo General Hospital, Western Uganda. Open J Nurs.

[42] Nsibu CN, Manianga C, Kapanga S, Mona E, Pululu P, Aloni MN. Determinants of Antenatal Care Attendance among Pregnant Women Living in Endemic Malaria Settings: Experience from the Democratic Republic of Congo. Hernandez E, editor. Obstet Gynecol Int [Internet]. 2016;2016:5423413. 10.1155/2016/5423413. 10.1155/2016/5423413 PMC5040812 27703482

[43] Chemouni B (2018). The political path to universal health coverage: power, ideas and community-based health insurance in Rwanda. World Dev.

[44] Njiku F, Wella H, Sariah A, Protas J. Prevalence and factors associated with late antenatal care visit among pregnant women in Lushoto, Tanzania. Tanzan J Health Res. 2017;19(3).

[45] Wagan F, Siyal AA, Ali R, Taqi T (2018). Major consequences, determinants and obstetrical outcomes of unintended pregnancy. Natl Editorial Advisory Board.

[46] Murugesan A, Sundaram R, Muthusamy M (2016). Awareness, attitude and practice of contraception among antenatal women in a tertiary care hospital-a cross-sectional study. Contraception.

[47] Alamneh A, Asmamaw A, Woldemariam M, Yenew C, Atikilt G, Andualem M (2022). Trend change in delayed first antenatal care visit among reproductive-aged women in Ethiopia: a multivariate decomposition analysis. Reprod Health.

[48] Ashimi AO, Amole TG (2015). Prevalence, reasons, and predictors for home births among pregnant women attending antenatal care in Birnin Kudu, Northwest Nigeria. Sex Reproductive Healthc.

[49] Joshi C, Torvaldsen S, Hodgson R, Hayen A (2014). Factors associated with the use and quality of antenatal care in Nepal: a population-based study using the demographic and health survey data. BMC Pregnancy Childbirth.

[50] Pandey S. Socioeconomic and Demographic Determinants of Antenatal Care Services Utilization in Central Nepal. Int J MCH AIDS (IJMA). 2013;2(2):212–9. PMC4948147 27621975

[51] Jacobs C, Moshabela M, Maswenyeho S, Lambo N, Michelo C (2017). Predictors of antenatal care, skilled birth attendance, and postnatal care utilization among Zambia’s remote and poorest rural communities: a multilevel analysis. Front Public Health.

[52] Mekonnen Y, Ayichiluhm M, Dejenu G (2015). Prevalence and determinants of home birth after AnteNatal Care attendance in Gozamin District, Northwest Ethiopia. Health Sci J.

